# Mapping the evolution of entrepreneurship as a field of research (1990–2013): A scientometric analysis

**DOI:** 10.1371/journal.pone.0190228

**Published:** 2018-01-04

**Authors:** Yanto Chandra

**Affiliations:** Department of Public Policy, City University of Hong Kong, Hong Kong SAR, China; Institut Català de Paleoecologia Humana i Evolució Social (IPHES), SPAIN

## Abstract

This article applies scientometric techniques to study the evolution of the field of entrepreneurship between 1990 and 2013. Using a combination of topic mapping, author and journal co-citation analyses, and overlay visualization of new and hot topics in the field, this article makes important contribution to the entrepreneurship research by identifying 46 topics in the 24-year history of entrepreneurship research and demonstrates how they appear, disappear, reappear and stabilize over time. It also identifies five topics that are persistent across the 24-year study period––institutions and institutional entrepreneurship, innovation and technology management, policy and development, entrepreneurial process and opportunity, and new ventures*–*–which I labeled as The Pentagon of Entrepreneurship. Overall, the analyses revealed patterns of convergence and divergence and the diversity of topics, specialization, and interdisciplinary engagement in entrepreneurship research, thus offering the latest insights on the state of the art of the field.

## Introduction

Entrepreneurship is a highly dynamic and fast growing scholarly field of research with a long intellectual tradition. Its intellectual roots can be traced back to the work of economists such as Cantillon [1], Smith [2], Knight [3], and Schumpeter [4], who laid the foundations by defining entrepreneurship and its relationship with innovation, economic growth and uncertainty. After a rather sluggish growth for decades, entrepreneurship research gained some momentum with an emphasis on the person-centric approach, which attributes psychological traits and people’s characteristics as predictors of entrepreneurship [5, 6]. As it evolved, the field experienced a behavioral turn, with growing emphasis on what entrepreneurs really do; particularly why and how they recognize, evaluate, and exploit opportunities [7]. Some scholars argued that the field had become fragmented, and criticized that the field became a broad label under which a ‘potpourri’ of research was housed [8]. Other scholars concluded that the field was highly permeable, relied heavily on major management journals, and lacked boundaries and new theories [9].

Like in the sciences and other fields of social sciences, there is a tradition among entrepreneurship scholars to pause to take stock of what has been done in the past and reflect on the future. Mapping and tracking the evolution of entrepreneurship research is central to our understanding of the institutionalization of entrepreneurship, assess its legitimacy, and identify alternate histories and future opportunities. The collective success of the science of entrepreneurship is vital, as it helps entrepreneurs, policy makers and global institutions understand the drivers, obstacles and rules that affect value creation, economic growth, resource allocation and policy agenda that shape societal well-being. A number of scholars have attempted to examine the domain of entrepreneurship field, map its intellectual structure, and assess its evolution (see [9, 10, 11, 12, 13, 14]). Unfortunately, the studies depict conflicting findings with some scholars concluding a maturing [10] and converging pattern [13] while others suggest lack of maturity and diverging patterns in the entrepreneurship research [11, 12, 15]. Although these studies made a significant contribution to what we know about entrepreneurship as a field, they tend to be based on older bibliographic materials (i.e., up to 2009), and used a single analytical approach, i.e., primarily co-citation relations analysis. Therefore, these do not represent well the more recent development in entrepreneurship research. Moreover, co-citation analysis is only one of the techniques used in scientometrics; it can be enhanced by newer techniques in scientometrics including topic mapping and overlay visualization analyses to deepen our understanding of the field.

Scientometrics, or also known as ‘science mapping’ [16, 17, 18], is often used in conjunction with information visualization [19, 20] and text mining [21, 22] to study a large body of bibliographic materials, as well as measuring various kinds of scientific activities, including investments in research and personnel. Scientometricians have combined various techniques from scientometrics, information visualization and text mining to study the evolution of various fields of sciences, from biology [21], chemistry/nanotechnology [23], informetrics and scientometrics [16, 24], to cognitive science [25]. For instance, Oldham and colleagues [21] used scientometrics to visually map synthetic organisms, cells and genomes that inform global policy debates on the governance of synthetic biology, and that help promote independent and transparent monitoring of developments in synthetic biology. Leydesdorff and Goldstone [25] used scientometrics to map the emergence, branching and merging of the field of cognitive science as an interdisciplinary field among psychology, linguistics, computer science, philosophy and the neurosciences, and demonstrated how it differs with the progression of artificial intelligence. However, these novel techniques and approaches have largely been confined to their own fields, with little or no interaction with entrepreneurship research. To address the knowledge gap, this article adopts the best practices from the recent advances in scientometrics to answer two questions: first, *How has the entrepreneurship as a field of research changed over time*?, and second, *What are the latest trends in terms of new and highly cited topics in the field*?

By applying three analytical tools in scientometrics––topic mapping, co-citation, and overlay visualization analyses––on bibliometric data from Web of Science and focusing on micro (i.e., word), meso (i.e., article) and macro (i.e., journal) levels of analysis, I identify 46 topics in the history of entrepreneurship (1990–2013), and demonstrate how they appear, disappear, reappear and stabilize over time. I also identify five topics that are persistent across the 24-year study period, that I labeled here as The Pentagon of Entrepreneurship: *institutions; innovation and technology; policy and development; entrepreneurial process* and *opportunity;* and *new ventures*. This study complements previous bibliometric studies of entrepreneurship research by revealing that the literature in the field has *converged* and *diverged* as demonstrated by the stabilization of certain topics and identification of communities of scholars; and the *diversity* of topics, *specialization* and *interdisciplinary* engagement. To my knowledge, this is the *first* paper that offers topic mapping and overlay visualization analyses to map the evolution of entrepreneurship research in a single study. In the next section, I describe the methodology and data, and discuss what the findings mean and their implications.

## Methods and materials

### Analytical approach

Scientometrics is a body of tools and techniques to integrate knowledge in a given field or body of literature using quantitative analysis and statistics to describe patterns of publication. It allows researchers to conduct ‘science mapping’ [26] to synthesize research findings, evaluate the research and publication performance of individuals and institutions, and to reveal the (intellectual, network, conceptual) structure and dynamics of scientific fields. Recent advances in scientometrics include information visualization and text mining techniques [17, 25, 27] that help researchers dig deeper into the bibliographic materials and visualizing them to enhance analysis. In this article, I used three complementary scientometrics techniques to examine the evaluation of entrepreneurship as a field of research. This approach follows scientometricians’ call for the use of multi-methods in scientometrics analysis––or so-called method triangulation. For instance, Wen and colleagues [28] applied three scientometrics techniques in their scientometrics research and argued that the use of triangulation “produces a more comprehensive picture than each method applied individually. The outcomes from the three different approaches can be associated with each other and systematically interpreted to provide insights into the complex multidisciplinary structure of a field” (p.724). Other scholars such as Lundberg and colleagues [29] argued that “triangulation of data sources and methods can strengthen the validity in a study by enabling comparisons of different descriptions and explanations of the phenomenon” (p. 586). Some scholars applied triangulation by combining different scientometrics techniques and software, such as Vantage Point versus NetDraw versus VOSviewer [30] or citation relations versus shared author keywords versus title word-cited reference co-occurrence [28]; using different types of data, such as funding information and co-authorship data [29]; as well as using one analysis as a baseline to show contrast with other analysis or ‘overlay mapping’ [31] under study.

First, I extracted the latent topics embedded in the bibliographic materials of interest and their evolution, using topic mapping technique. *Topic mapping analysis* applies statistical procedures to turn latent (or hidden, invisible) topics in large bibliographic materials into explicit visuals that show the clusters of topics and the connections among them. Topic mapping (or topic community clustering) analysis is an emerging technique used in text mining and scientometrics [32, 33]. Topic modeling relies on the dissimilarities between two probability distributions: that is, the distribution of a semantic unit over the set of all topics, and the distribution of all semantic units together over the set of all topics [34]. When the two distributions are very dissimilar, it means that a semantic unit is likely to represent a domain-specific concept; but if the distributions are very similar, it means that a semantic unit does not represent a specific concept. The relationship among terms is counted by the number of times they co-occur across all articles. Thus, the larger the number of articles in which two terms co-occur, the stronger is the relationship between the two terms. Based on the relationships of terms, terms are grouped together into clusters and a map is constructed. This concept is called *visualization of similarities* or VOS [18, 34] and is a variant of the *community detection* algorithms developed by Clauset and colleagues [35] and Newman and Girvan’s [36] modularity measures of community structures.

**Fig 1:**
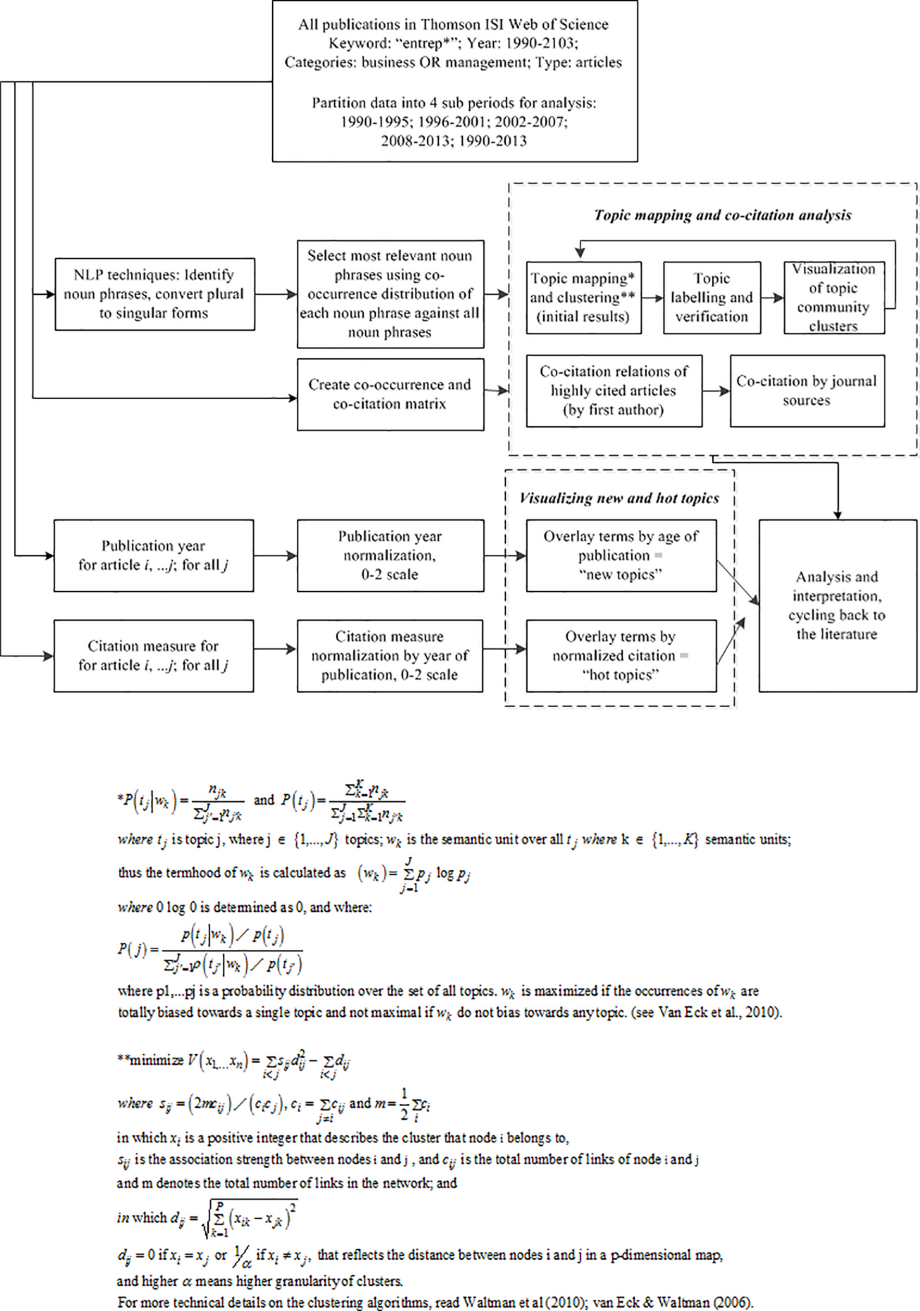
Design of the scientometric analysis.

To perform topic mapping, I started by using natural language processing (NLP) techniques to parse the titles and abstracts of the 3693 articles included in this study (see the Data Preparation section). This process yielded a list of all the nouns and sequences of nouns and adjectives that occurred in the articles. Following van Eck and colleagues’ [34, 37] Java-based VOSviewer procedures, only noun phrases that occurred in at least 10 articles were considered in the analysis. I developed a thesaurus to filter out “noise” information, such as *general noun* words (e.g., “study”, “implications”, “introduction”) and *articles* (e.g., “the”, “a”, “an”), *modal* words (e.g., “can”, “will”, “should”), *pronouns* (e.g., “I”, “we”, “they”), and publishing-related words (e.g., “Elsevier”, “Palgrave”, “copyright”). I also converted all plural nouns into singular nouns. From here, I created co-occurrence networks, and selected the most relevant terms or words (i.e. noun phrases) (see Fig 1 for the research design) and generated the topics from the based on their similarities.

I subsequently validated the topic mapping results using co-citation analyses at the author and journal levels and using overlay visualization analyses. *Co-citation analysis* is a statistical technique that can transform latent relationships among authors and or journals into explicit visual outputs in the form of co-citation clusters, to ease data interpretation. Co-citation analysis is one of the most popular techniques used in the bibliometric study in various business disciplines, from strategic management [38], business ethics [39] to international business [40]. The idea behind co-citation analysis is that the articles of scholars who are frequently co-cited are likely to represent similar or related concepts [38, 41]. I used co-citation analysis [41, 42] to provide further insights and validate the topic mapping results above. To do this, I created a co-citation matrix and used Van Eck’s [43, 44] clustering technique (see Fig 1) to identify the clusters of closely related publications as “topics”. Using Van Eck’s Java-based VOSviewer [18, 43, 45] techniques, I conducted co-citation relations of articles with a minimum of 20 citations. The purpose of using the “20-citations threshold” was to reduce clutter in the data visualization and this was found to provide cleaner and less cluttered visuals compared to using lower citation threshold (e.g., at 5, 10 or 15 citations). The co-citation analysis was conducted at the *author* and *journal* levels. The former calculates co-citation based on the relations of authors’ of articles, while the latter on journal sources’ relations. The author- and journal-based co-citation relations served to offer richer insights into the intellectual structure of entrepreneurship.

*Overlay visualization analysis* detects the latest topics (“new topics”) and the topics that appeared in highly cited journals (“hot topics”), which enables researchers to portray the trajectory of a research field. Overlay visualization is one of the most cutting-edge techniques used in scientometrics and information visualization [37, 46] to display publication trends. It provides a “visual history” of a field of research. Based on a thorough review of the literature, this technique has not been used in prior bibliometric study in entrepreneurship. Using Van Eck’s Java-based VOSviewer techniques [37, 45], I plotted a base map based on the relationships between a type of element (e.g., terms relations that form clusters of topics), and then *overlay* each data point with additional numerical information that adds value for interpretation (e.g., age of publication, citation impact, etc.). In this study, I used two types of overlay visualization to depict publication trends: *time* and *citation*.

Although triangulation of methods is critical to achieve rigor and consistency in a scientometric study, the three-pronged approach used in this article (i.e., topic mapping, co-citation and overlay visualization) has not been used in the previous scientometric research on entrepreneurship. The three scientometrics techniques used in this article was driven by their complementarity where additional insights and validity are gained by comparing different techniques [28,29]. Specifically, topic mapping provides a synthesis of the themes using words used in the published articles, co-citation analysis offers insights on the relationships among authors and journals as a proxy to identify research themes and networks, while overlay visualization analysis generates the newest and hot topics–thus the combination of the three analytical techniques provides a more well-rounded view of the findings at the word, co-citation, year and citation rate levels and allows the verification of findings generated by each technique (than using a single analytical technique). More details of how each of the analytical techniques was used will be discussed in more details in the Findings section.

### Data preparation

To perform scientometric analysis of entrepreneurship as a field of research, I used data from the Clarivate Analytics’ Web of Science (WoS) Core Collection database. This database is commonly used in scientometrics to study the progress and evaluation of various scientific fields [43,47]. The data collection took place in June 2014, and therefore the database included data up to the end of 2013. Before the actual data collection on WoS, I conducted preliminary observation of the database. I found that there were few entrepreneurship papers published in journal articles prior to 1990, with the period of 1990–1995 yielding only 121 articles. I am also mindful of the fact that topic mapping analysis, one of the key analytical techniques used in this study, produces better results with larger bibliometric collections. Thus, 1990 was chosen as the cut-off point. A Web of Science search using “entrep*” keyword (following [10, 11]) and screening for “articles” only for the Business OR Management subject categories within the 1990–2013 period produced 3693 publications for analysis. Although engineering, science and arts/humanities literature also contains research on entrepreneurship, to date the field of entrepreneurship remains a core area of research in the business and management domains. Therefore, the bibliographic samples were focused on the “business OR management” categories to make a contribution to the domains that gave birth to entrepreneurship. Only journal articles were chosen because journal articles are “certified knowledge” [48]. Next, I parsed the publications’ Abstract & Title into a whole 1990–2013 corpus and four separate sub corpora for finer analysis: 1990–1995, 1996–2001, 2002–2007, and 2008–2013. Although there is no strict formula on the range or intervals of bibliometric data used in a scientometric analysis (some scholars use 10-, 7- or 5-year intervals), this data parsing was reasonable and eased the detection of changes in the publication trends. The design for the study is summarized in the Fig 1.

## Results

As shown in Fig 1, the distribution of entrepreneurship articles published across the four periods is as follows: 1990–1995 (*n* = 121 articles), 1996–2001 (*n* = 262), 2002–2007 (*n* = 866), and 2008–2013 (*n* = 2444). As can be seen in the distribution of the bibliographic materials, there is a sudden explosion in the number of entrepreneurship articles in recent years and the largest increase in publication took place in the last two periods.

Some of the most highly cited publications in this analysis are (as of the date of data collection): 1) Shane and Venkataraman’s [7] “The Promise”, an Academy of Management Review paper (Rank #1: 1578 citations); 2) Shane’s [49] “Prior Knowledge”, an Organization Science paper (Rank #2: 805 citations); 3) Zott and Amit’s [50] “E-business”, an Strategic Management Journal paper (Rank #7: 587 citations).

To provide an overall picture of the evolution of entrepreneurship as a field of research (1990–2013), I conducted three complementary analyses: topic mapping, co-citation and overlay visualization analyses.

### Topic mapping analysis

I began the analysis by performing topic mapping using Van Eck’s Java-based VOSviewer techniques [18, 37, 43, 45] using the four periods of bibliometric data, and I then sought to give a label to all topic clusters that emerged in each interval according to the terms and phrases that were prominent in each period and depict the number of terms (i.e., words) in each topic cluster and calculated their share out of all terms in each period. Another scholar with expertise in scientometrics and entrepreneurship played a “devil’s advocate” role to re-examine the topic labels to ensure that all labels make logical sense. Each term had appeared in at least 10 publications/articles. I offered a summary of the overall topic clusters and their share (using VOSviewer techniques) over the four periods in Table 1. The breakdown of the topic clusters in the Table 1 is shown in S1–S6 Figs.

**Table 1:** Topic clusters of entrepreneurship research 1990–2013 (n = 3693).

topic cluster #	topic community clusters	top 5 terms	2008–2013		2002–2007		1996–2001		1990–1995	
			#terms	share	#terms	share	#terms	share	#terms	share
1	person-centric	person, attitude, belief, psychology, self efficacy	648	5.80%	212	5.73%	533	36.96%	229	46.26%
2	performance	performance, firm performance, manager, environment, profitability	480	4.30%	265	7.17%	87	6.03%	117	23.64%
3	new venture creation	startup, founder, new venture, entry, founding	783	7.01%	189	5.11%	66	4.58%	86	17.37%
4	innovation & tech management	technology transfer, commercialization, innovation, R&D, patent	1057	9.46%	225	6.09%	331	22.95%	63	12.73%
5	entrepreneurial process and opportunity	entrepreneurial process, opportunity, emergence, evolution, case study	875	7.83%	251	6.79%	172	11.93%	0	0
6	failure	failure	0	0.00%	26	0.70%	24	1.66%	0	0
7	strategy	corporate entrepreneurship, competitive advantage, strategy, dynamic capability	418	3.74%	86	2.33%	101	7.00%	0	0
8	experience and knowledge	experience, knowledge	shifted to #38*	0.00%	0	0.00%	61	4.23%	0	0
9	network	social capital, network, social network	shifted to #3*	0.00%	63	1.70%	25	1.73%	0	0
10	culture	culture	shifted to #44*	0.00%	0	0.00%	18	1.25%	0	0
11	smes	owner, smes, small business, small business management	shifted to #16*	0.00%	172	4.65%	24	1.66%	0	0
12	institutional entrepreneurship	institution, agency, identity, institutional theory, institutional entrepreneurship	1194	10.68%	265	7.17%	0	0	0	0
13	narrative and discourse	discourse, meaning, narrative, story	shifted to #31*	0.00%	64	1.73%	0	0	0	0
14	community and society	society, community, learning	0	0.00%	130	3.52%	0	0	0	0
15	ethics	ethics	shifted to #32*	0.00%	17	0.46%	0	0	0	0
16	internationalization and intl entrepreneurship	internationalization, intl entrepreneurship, smes, small firm, intl new venture	741	6.63%	75	2.03%	0	0	0	0
17	market orientation	marketing, market orientation	31	0.28%	35	0.95%	0	0	0	0
18	entrepreneurial orientation	entrepreneurial orientation, autonomy, strategic orientation, proactiveness	194	1.74%	24	0.65%	0	0	0	0
19	venture capital	venture capitalist	shifted to #4*	0.00%	15	0.41%	0	0	0	0
20	entrepreneurial behavior	behavior, propensity, competency, entrepreneurial behavior, attitude	shifted to #5*	0.00%	115	3.11%	0	0	0	0
21	decision making and risk	decision, risk, profit	0	0.00%	125	3.38%	0	0	0	0
22	investment, capital and financing	start, investment, capital, financing	shifted to #4*	0.00%	178	4.81%	0	0	0	0
23	human capital	human capital, scientist, advantage, engineering, internet	164	1.47%	35	0.95%	0	0	0	0
24	competition	competition	0	0.00%	28	0.76%	0	0	0	0
25	initial public offering	sale, firm sale, initial public offering, ipo	18	0.16%	38	1.03%	0	0	0	0
26	policy and develoment	regional development, policy, region, government, economic growth	895	8.01%	362	9.79%	0	0	0	0
27	family business	family firm, family, family business, ownership, ceo	324	2.90%	60	1.62%	0	0	0	0
28	entrepreneurship education	education, student, training, entrepreneurship theory, perception	451	4.04%	357	9.66%	0	0	0	0
29	female entrepreneurship and gender	women, self employment, gender, women entrepreneur, labour market	397	3.55%	175	4.73%	0	0	0	0
30	ownership and stakeholders	ownership, employee, barrier, stakeholder	0	0.00%	110	2.98%	0	0	0	0
31	social entrepreneurship	social entrepreneurship, discourse, construction, social enterprise, social value	290	2.60%	0	0	0	0	0	0
32	business ethics	business ethics, tension, ethic	88	0.79%	0	0	0	0	0	0
33	corporate social responsibility	corporate social responsibility, business school, social responsibility	46	0.41%	0	0	0	0	0	0
34	leadership	transformation, leadership, principle, leader, cognition	214	1.91%	0	0	0	0	0	0
35	poverty, norm and tradition	norm, poverty, tradition	92	0.82%	0	0	0	0	0	0
36	employment and job creation	employment, population, global entrepreneurship monitor, income, economic activity	670	6.00%	0	0	0	0	0	0
37	human resources management	human resources management, human resource, chinese firm, hrm	75	0.67%	0	0	0	0	0	0
38	capability, exploration and exploitation	capability, team, exploitation, exploration, prior knowledge	437	3.91%	0	0	0	0	0	0
39	orientations	orientation, innovativeness, discovery, novely, risk taking	252	2.26%	0	0	0	0	0	0
40	value creation	value creation, conceptual model	68	0.61%	0	0	0	0	0	0
41	multinational enterprises and business strategy	corporation, business strategy, multinational corp, subsidiary, mnc	92	0.82%	0	0	0	0	0	0
42	academic entrepreneurship	academic entrepreneurship, reputation	51	0.46%	0	0	0	0	0	0
43	intrapreneurship	intrapreneurship	15	0.13%	0	0	0	0	0	0
44	entrepreneurial culture	entrepreneurial culture	12	0.11%	0	0	0	0	0	0
45	governance and board of directors	governance, board, director	86	0.77%	0	0	0	0	0	0
46	top management team	top management team	17	0.15%	0	0	0	0	0	0
			11175	100%	3697	100%	1442	100%	495	100%

In the first period, 1990–1995, four topic clusters emerged (see Table 1 and S1 Fig). Topic mapping analysis (S1 Fig) revealed four topic clusters of entrepreneurship research: 1) *person-centric* (key terms: “person”, “ability”; red circles); 2) *performance* (key terms: “performance”, “environment”; green circles); 3) *new venture* (key terms: “new venture”, “management”; blue circles); and 4) *innovation and technology* (key terms: “innovation”, “technology”; yellow circles). During this period, entrepreneurship research was dominated by person-centric topics (accounting for nearly half of the articles analyzed), followed by performance and new venture creation.

The second period, 1996–2001, was marked by various publications that attempted to define the field, and by self-reflective papers [7, 8]. The topics in this period had increased to 11 clusters (see Table 1 and S2 Fig): 1) *person-centric* (red circles); 2) *performance* (green circles); 3) *new venture* (blue circles); 4) *innovation and technology* (yellow circles); 5) *opportunity and entrepreneurial process* (purple circles); 6) *failure* (light blue circles); 7) *strategy and capability* (navy blue circles); 8) *experience and knowledge* (amber circles); 9) *network* (orange circles); 10) *culture* (pink circles); and 11) *small business* (brown circles). During this period the *person-centric* approach in entrepreneurship continued to occupy the largest share (see Table 1), but there was a sudden growth in *innovation and technology management* topics, and the rise of *entrepreneurial process and opportunity* topics. Other emerging topics were entrepreneurial failure, strategy, culture, and network.

The third period, 2002–2007, was characterized by a sudden explosion in the number of topics and the number of articles published. There were several interesting patterns observed. Topic mapping analysis revealed 28 clusters (see Table 1 and S3 Fig). The *person-centric* topic decreased substantially, while *entrepreneurial process and opportunity*, *SMEs*, *new venture creation* and *performance* increased substantially. The topics *strategy* and *innovation and technology management* seemed to stabilize. This period saw the emergence of 19 additional topics that were not strongly present in the first and second periods (as shown by their topic cluster number and color code): 12) *institutional entrepreneurship* (jade blue circles); 13) *discourse and narrative* (hot pink circles); 14) *community and society* (dark purple circles); 15) *ethic* (light purple circles); 16) *internationalization and international entrepreneurship* (grey circles circles); 17) *marketing and market orientation* (emerald green circles); 18) *entrepreneurial orientation* (baby blue circles); 19) *venture capital* (lime green circles); 20) *entrepreneurial behavior* (plum circles); 21) *decision making and risk* (copper brown circles); 22) *investment and financing* (moss green circles); 23) *human capital* (bright purple circles); 24) *competition* (light brown circles); 25) *IPO and firm sale* (lavender circles); 26) *policy and development* (bright green circles); 27) *family business* (purple circles); 28) *entrepreneurship education* (light purple circles); 29) *self-employment and women entrepreneurship* (grey circles); and 30) *ownership and stakeholders* (bright purple circles).

**Fig 2:**
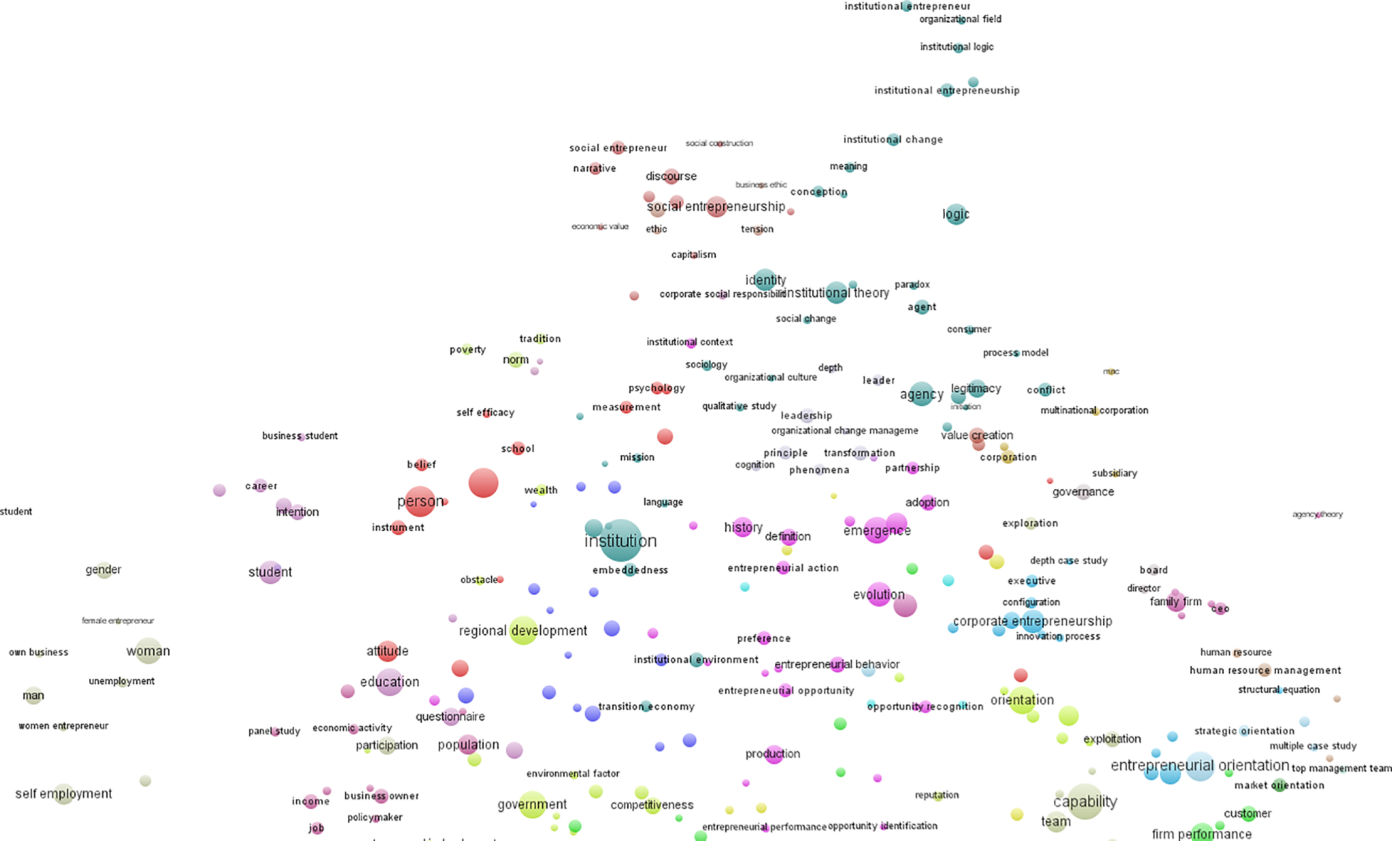
Topic clusters of entrepreneurship research 2008–2013 (n = 2444), top half.

**Fig 3:**
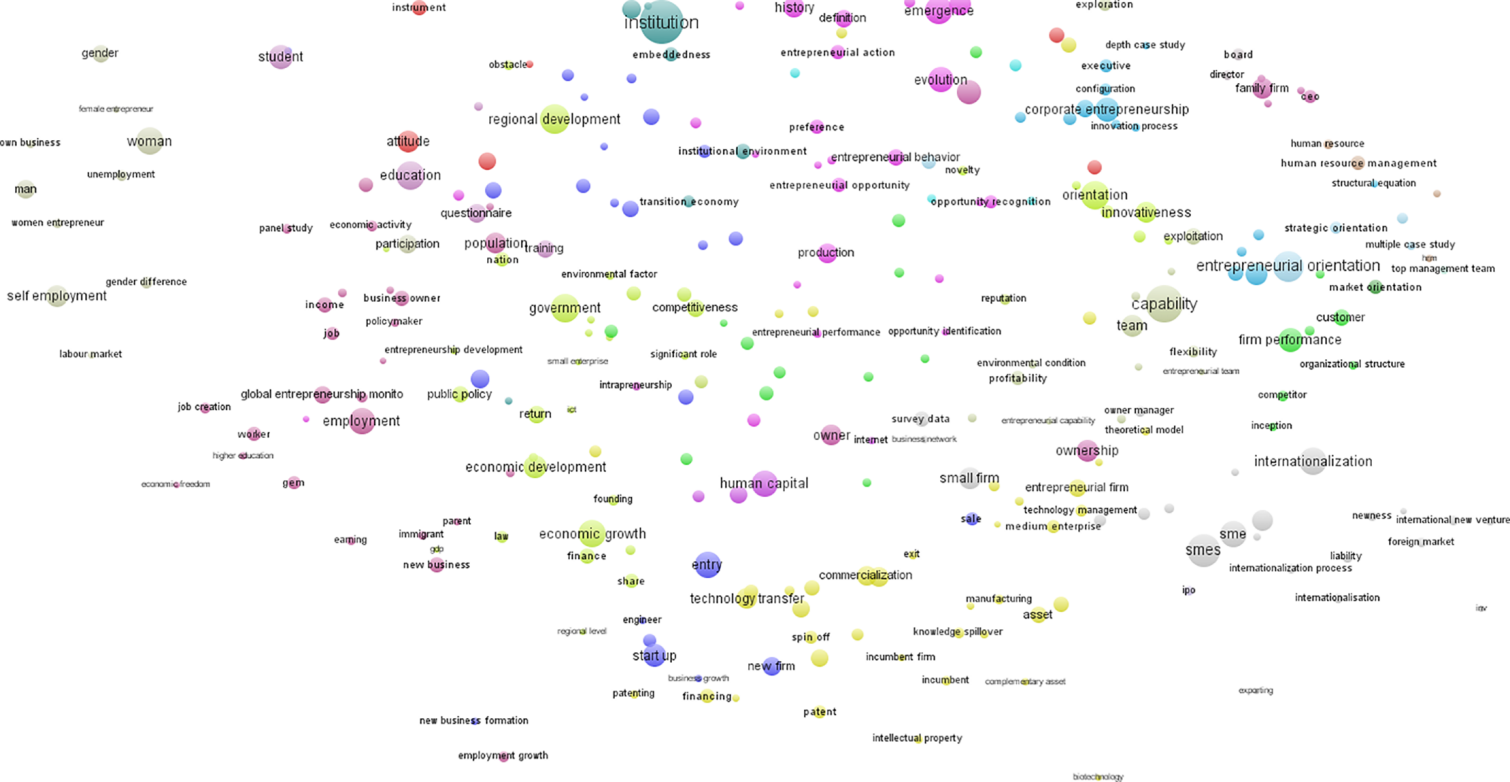
Topic clusters of entrepreneurship research 2008–2013 (n = 2444), bottom half.

The fourth period, 2008–2013, revealed that some topics disappeared and some became a part of other topics (based on their proximity on the 2-dimension map), and some new topics emerged. In total, there were 32 cluster topics in this period, out of a total of 46 that existed between 1990 and 2013 (see Table 1 and Fig 2 and Fig 3). The person-centric topic has stabilized and captured a persistent share, albeit with a greater number of articles. There were sudden increases on these topic clusters: *entrepreneurial process and opportunity*, *innovation and technology management*, *new venture creation*, *strategy*, *entrepreneurial orientation*, *internationalization and international entrepreneurship*, *human capital*, *family business*, and *female entrepreneurship and gender*. But the most notable increase was with the emergence of *institutional entrepreneurship* (which captured a 10% share of all terms), *innovation and technology management*, and *policy and development*. A number of very new topics that emerged in this period were: *social entrepreneurship*, *business ethics*, *corporate social responsibility*, *leadership*, *poverty*, *norm and tradition*, and *employment and job creation*. Topics that seemed to weaken included *competition*, *failure*, and *decision making and risk*.

### Co-citation analysis

I conducted co-citation analysis at the author and journal levels, as described further below.

#### Author-based co-citation analysis

**Fig 4:**
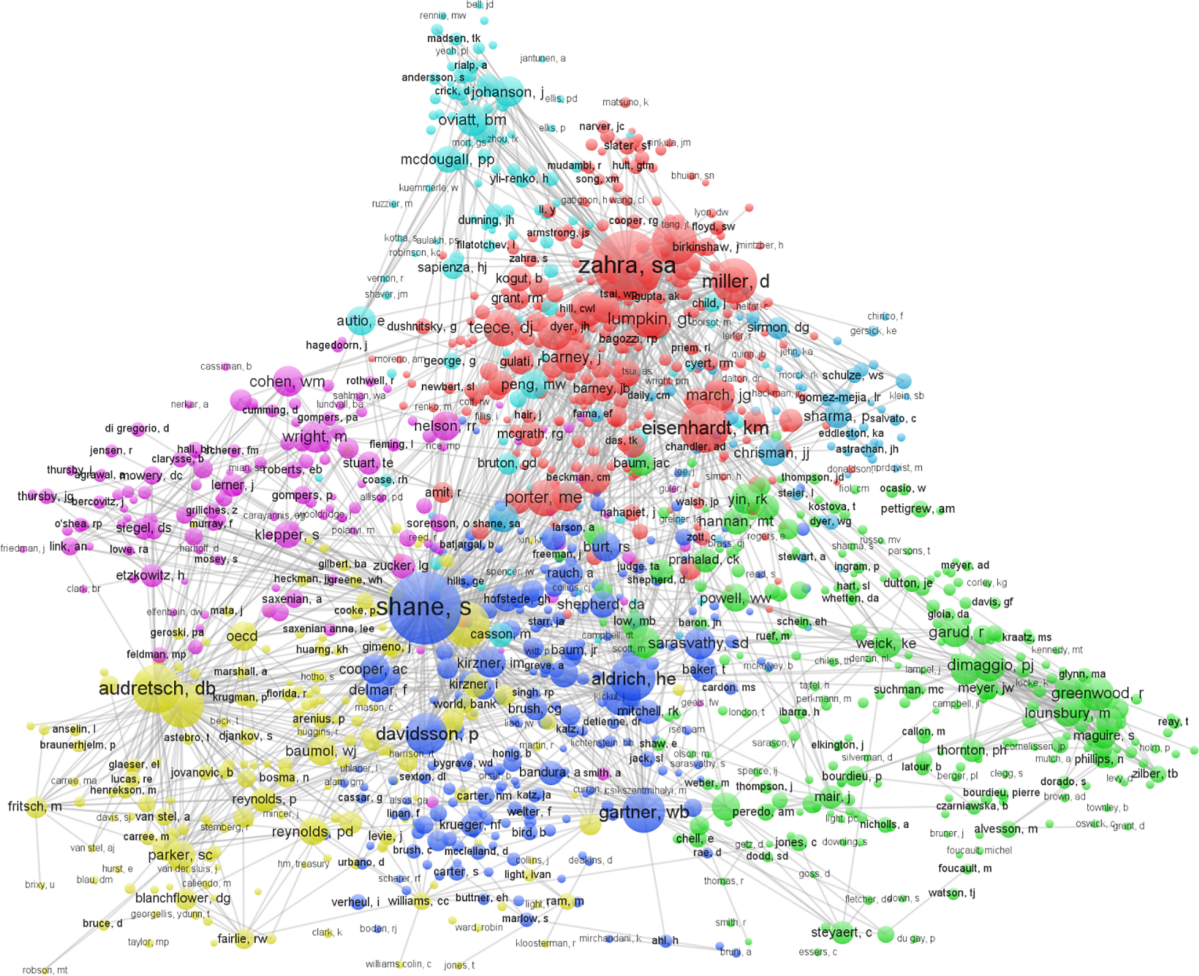
Author co-citation clusters in entrepreneurship research 2008–2013 (by first author; citation ≥ 20).

I conducted co-citation analysis of authors who were co-cited across the four periods to offer a more holistic interpretation of the evolution of the field. In the analysis, I included only articles that had 20 or higher citations and included the name of the “first author” only to avoid overly cluttered maps, following the procedures suggested by Rodrigues et al. [45] and Waltman and Van Eck [43]. Fig 4 shows the co-citations for the period of 2008–2013, while S4–S6 Figs show the co-citations for the other three periods. Larger node and node labels reflect higher citations (and vice versa), while different color and adjacent nodes depict the clusters of topic themes that emerge.

The results show that two major clusters of author co-citation relations emerged in the 1990–1995 period (see S4 Fig): *entrepreneurship-psychology* (Gartner, Cooper, Birley, Brockhaus, Aldrich, and Hannan; green circles) and *strategy-general management* (Burgelman, Kanter, Porter, Mintzberg, Drucker, Miller, McMillan; red circles). These co-citation clusters resembled the four clusters identified in the topic mapping analysis above, although they were less refined (see S1 Fig and column 7 of Table 1).

Next, author co-citation relations in the 1996–2001 period revealed four co-citation clusters (see S5 Fig): *entrepreneurship-innovation-psychology* (Gartner, Cooper, MacMillan, Timmons and the group; red circles); *strategy-innovation* (Covin, Zahra, Dess, Miller, Mintzberg, Burgelman, Stevenson; green circles); *strategy-economics* (Porter, Eisenhardt, Williamson, Tushman, Barney and the group; blue circles); and *organization-technology-innovation* (Carroll, Hannan, Acs, Westhead, Storey, Rothwell, Shane and the group; yellow circles). In this period, new highly cited scholars emerged: Covin, Zahra, Miller, Williamson, Eisenhardt, Venkataraman, and Shane. These co-citation clusters resembled the 11 clusters identified in the topic mapping analysis above, although they were less refined (see S2 Fig and column 6 of Table 1).

The author co-citation relations in the 2002–2007 period revealed six co-citation clusters (see S6 Fig): *entrepreneurship-psychology* (Shane, Gartner, Cooper, Johannisson, Busenitz, Davidsson, Baron, McClelland, Venkataraman, Sarasvathy and the group; red circles); *economics—innovation* (Audretsch, Baumol, Evans, Reynolds, Acs, Storey, Kirzner, Teece, Nelson and the group; blue circles); *institutions—network—technology—innovation—sociology* (Aldrich, Dimaggio, Greenwood, Burt, Powell, Garud, Hannan, Tushman, Van de Ven and the group; yellow circles); *international—entrepreneurship* (McDougall, Oviatt, Knight, Johanson, Cavusgil; purple circles), *strategy—technology—organization* (Zahra, Miller, Covin, Lumpkin, Porter, Eisenhardt, Barney, Burgelman, Von Hippel, Alvarez and the group; green circles); and *venture capital—finance—family business—cognition* (Westhead, Shepherd, Wright, Jensen, Chrisman, Sharma, Ensley and the group; light blue circles). Among the most highly cited scholars in this period were Shane, Zahra, Aldrich, Gartner, Eisenhardt, Audretsch, McDougall, Oviatt, Baron, and Dimaggio. Those who emerged in this period but did not feature in the prior two periods was McDougall. These co-citation clusters resembled the 28 clusters identified in the topic mapping analysis above, although they were less refined (see S3 Fig and column 5 of Table 1).

The author co-citation relations in the 2008–2013 period, as shown in Fig 4, revealed very dense clustering patterns compared to the previous three periods (see S4–S6 Figs). Nine co-citation clusters emerged in this period: *innovation—technology—venture—capital—institution* (Klepper, Cohen, Lerner, Nelson, Zucker, Stuart and the group; purple circles); *economics—innovation—networks* (Audretsch, Acs, Parker, Baumol, Fritsch, Arenius, Bosma, Minniti, North, and the group; yellow circles); *entrepreneurship—psychology—cognition—sociology—women* (Shane, Gartner, Aldrich, Davidsson, Baron, Bandura, Sarasvathy, Baker, Shepherd, Busenitz, Kirzner, Brush, Krueger, and the group; blue circles); *institution—organization—innovation—sociology—network* (Dimaggio, Greenwood, Weick, Garud, Powell, Johannisson, Suddaby, Dorado, and the group; green circles); *social entrepreneurship—narrative—education* (Mair, Steyaert, Hjorth, Austin, Nicholls, Tracey, Dees, Jones, Chell, Peredo, and the group; also green circles), *family business—strategy* (Chrisman, Sharma, Sirmon, Schulze, and the group; teal blue circles); *strategy—networks—capabilities—exploration* (Zahra, Eisenhardt, Miller, Covin, Lumpkin, Teece, Barney, Kogut, McGrath and the group; red circles); *marketing* (Slater, Kohli, Narver, Day, Atuahene-Gima, Zhou, and the group; also red circles); and *international—entrepreneurship* (McDougall, Oviatt, Coviello, Johanson, Dunning, Jones, Knight, Madsen, Cavusgil, and the group; light blue circles).

As shown above, results from co-citation analysis at the *author* level alone provided rather crude clusters of topics and offered overlapping topics, and the results can be rather difficult to interpret and depend on the researcher’s subjectivity in classifying and labeling them into topics. Nevertheless, they did show themes that offered support to the topic mapping results.

#### Journal-based co-citation analysis

**Fig 5:**
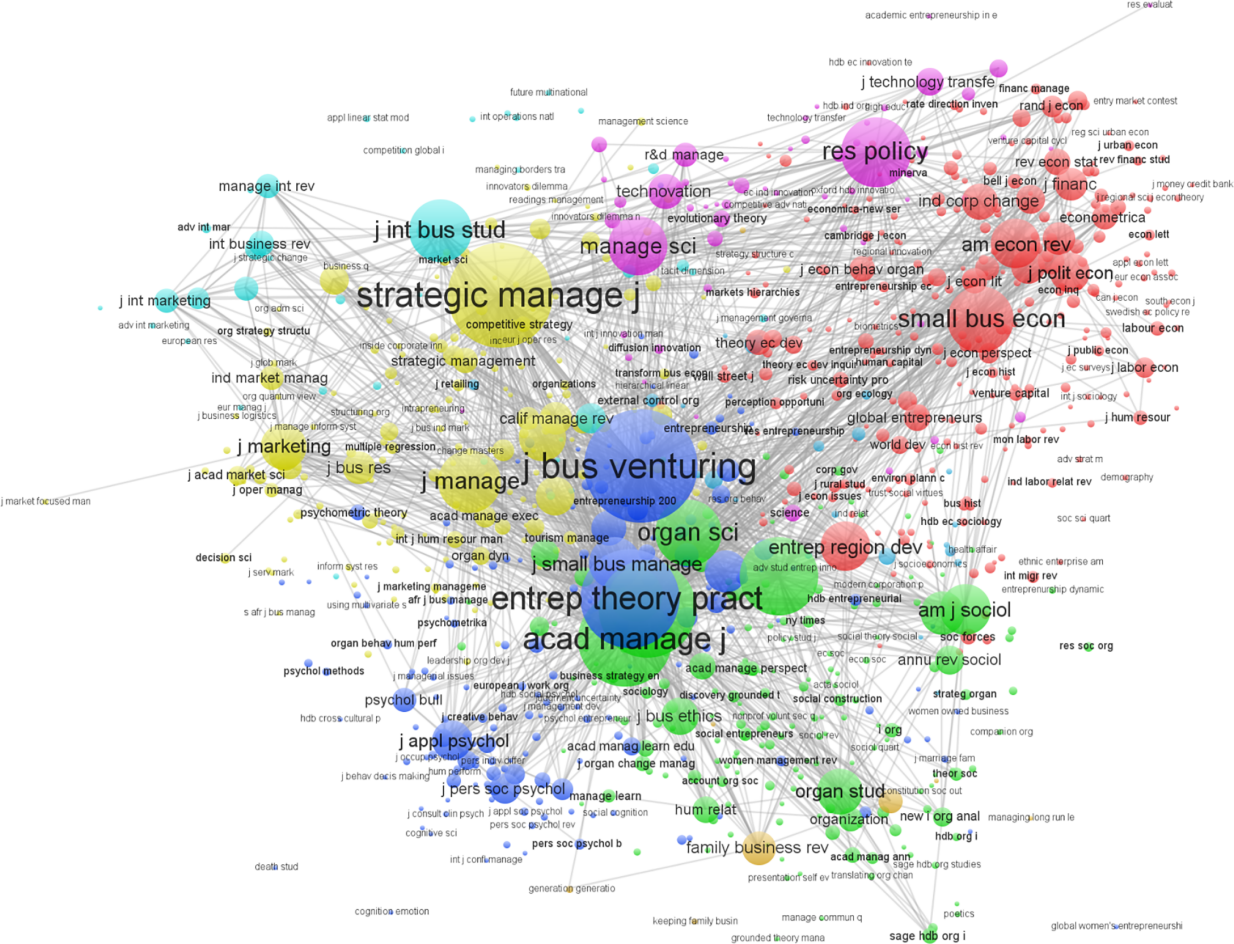
Journal-based co-citation clusters in entrepreneurship research 1990–2013 (by journal sources; citation ≥ 20).

To add more depth to the analysis, I conducted co-citation analysis based on journal sources of articles that had 20 or more citations (*n* = 945 articles) for the entire 1990–2013 period, using Van Eck’s Java-based VOSviewer techniques [33, 43]. Results are shown in Fig 5. The figure depicts a rather diverse and complex journal co-citation clusters: *entrepreneurship—psychology* (Journal of Business Venturing, Entrepreneurship Theory and Practice, Journal of Applied Psychology, Journal of Personality and Social Psychology; blue circles); *management—organizations* (Academy of Management Journal, Academy of Management Review, Administrative Science Quarterly, Organization Science; green circles); *family business* (Family Business Review; brown circles); *economics—finance* (Small Business Economics, American Economic Review, Entrepreneurship and Regional Development, Journal of Finance; red circles); *technology—innovation* (Research Policy, Management Science, Technovation, R&D Management; purple circles); *strategy—management* (Strategic Management Journal, Journal of Management, Harvard Business Review; yellow circles); *international business—entrepreneurship* (Journal of International Business Studies, International Business Review, Journal of International Marketing; light blue circles); and *marketing—innovation* (Journal of Marketing, Journal of Product Innovation Management, Industrial Marketing Management; yellow circles). The patterns of journal co-citations were not surprising, and resembled the more refined topic mapping results shown in Table 1.

#### Overlay visualization analysis of new and hot topics

**Fig 6:**
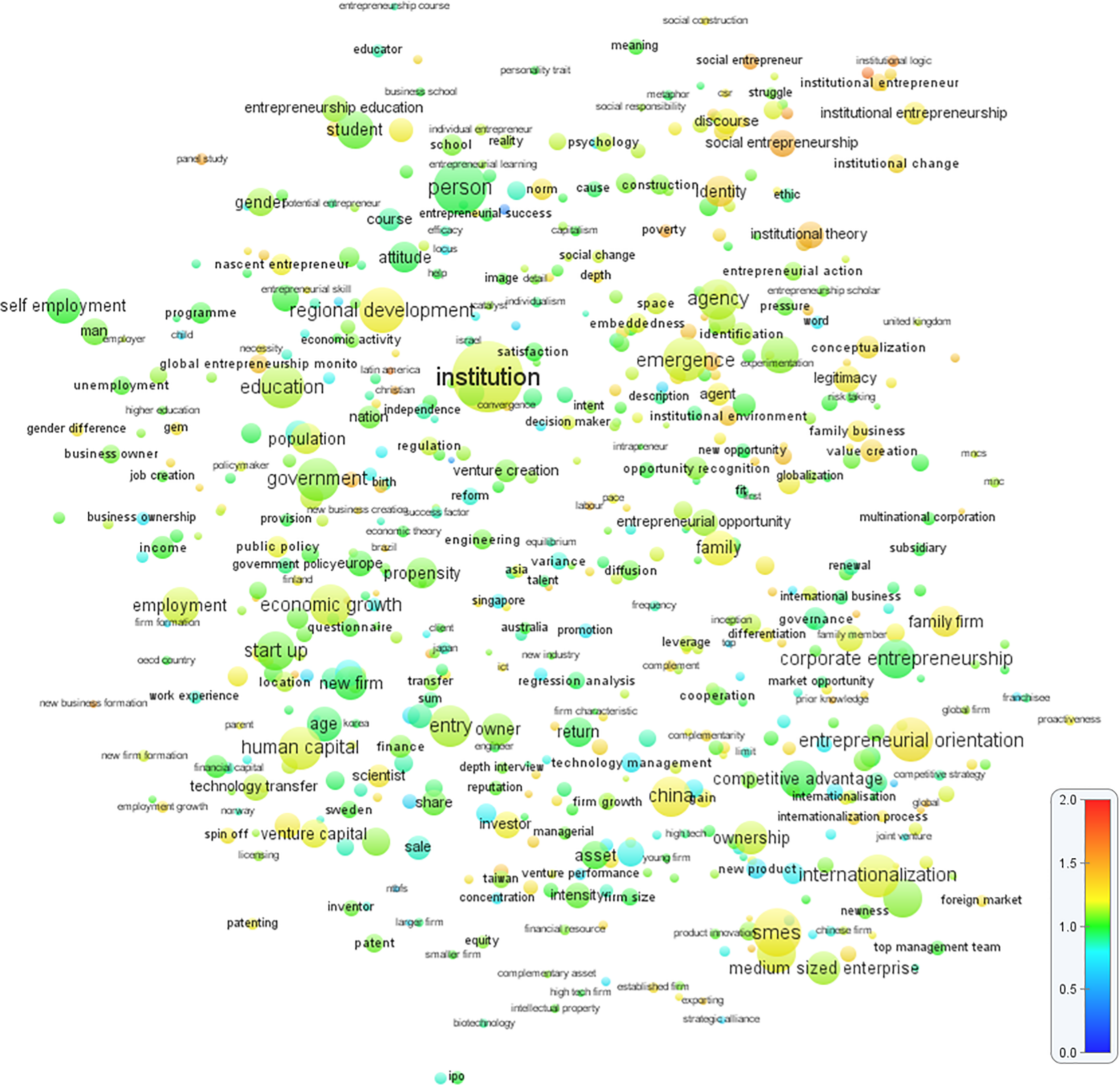
Overlay map of “new topics” in entrepreneurship research (1990–2013). The closer two terms are to each other, the stronger their relations. A normalized scale of 0–2 was used to indicate the newness of publications. Year 2008 was used as the mid-point (score 1). Terms that are used more towards 2013 are shown in orange to red, while terms that are used more towards 1990 are shown in light to dark blue. Each term occurs in at least 10 publications.

First, to produce “new topics” using the *time-based overlay visualization*, I plotted the entire topic mapping’s terms (or words) and clusters from 1990–2013, and I overlaid the base map with numerical information to depict new topics (and later, hot topics) in entrepreneurship research. I chose the year 2008 as the average midpoint at 1.0 of the scale (green). To visualize the new topics, the terms that appeared in the topic clusters were matched with the corresponding year of the article where the terms appeared. Newer topics were visualized using color ranging from yellow (relatively new) to red (the newest), while older topics were visualized from green (relatively old) to blue (the oldest), based on a normalized scale of 0–2. Thus, terms that were used more towards 2013 were shown in orange to red; while terms that were used more towards 1990 were shown in light to dark blue. This produces a color-based visualization of newer versus older publications. The result is shown in Fig 6, and the classification of topic clusters refers to the Table 1. Increasing trends in publications related to the following “new topics” were observed: *institutional entrepreneurship*, *institutional logic*, *institutional theory* (topic cluster #12), *social entrepreneurship*, *narrative*, *discourse* (topic cluster #31), *poverty* (topic cluster #35), *business ethics* (topic cluster #32), *family business* (topic cluster #27), *internationalization and international entrepreneurship* (topic cluster #16), and *global entrepreneurship monitor and use of panel data* (unclassified cluster).

**Fig 7:**
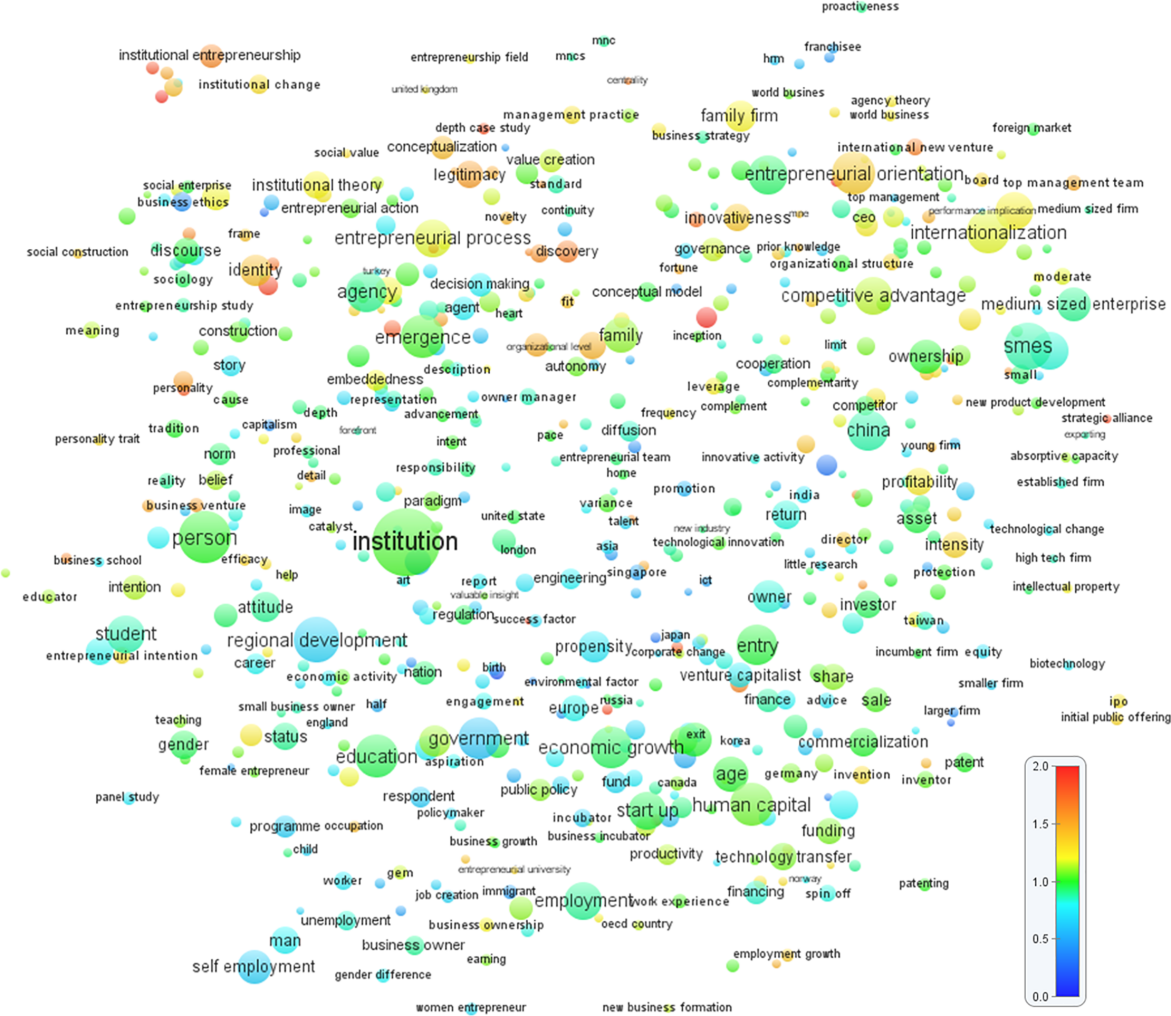
Overlay map of *overall* “hot topics” in entrepreneurship research (1990–2013). The closer two terms are to each other, the stronger their relations. The size and color of a term indicates, respectively, the number of publications in which the term occurs and the average citation impact of these publications. A normalized scale of 0–2 was used to indicate the average citation impact of publications. Blue indicates a low citation impact, green a normal citation impact, and red a high citation impact. Each term occurs in at least 10 publications.

Next, to produce “hot topics” using the *citation-based overlay visualization*, I plotted the terms (following [37]) with colored circles to reflect the average citation impact for the term. To visualize the hot topics (i.e., topics that appear in highly cited articles), I matched the terms that appeared in the topic clusters with the citation score of the article where the terms appeared. I corrected for the age of publications by dividing each publication’s number of citations by the average number of citations of all publications that appeared in the same year. This yielded a publication’s normalized score. Thus, a score of 1 means that a publication’s number of citation equals the average of all publications that appeared in the same field in the same year. The normalized citation scores of all publications in which the terms occurred were then averaged, after which a color scale that ranged from blue (0; the coldest) to green (midpoint of 1.0; relatively cold) and yellow (1; relatively hot), to red (2; the hottest) was used to plot the terms. Therefore, terms with a low average citation impact were marked blue, while terms with a high average citation impact were red. This produces a color-based visualization of hot (highly cited) versus cold (less cited) publications. The result is shown in Fig 7, and the classification of topic clusters refers to the Table 1. The “hot topics” included: *institutional work*, *institutional logic*, *institutional entrepreneurship* (topic cluster #12), *opportunity discovery and recognition* (topic cluster #5), *international new venture*, *international business study* (topic cluster #16), *entrepreneurial orientation*, *innovativeness* (topic cluster # 18), *cognition*, *emotion*, *identity* (a mix of topic clusters #1 and #34), *top management team* (topic cluster #46), *strategic alliance* (overlap of topic clusters #4 and #11), *performance and profitability* (topic cluster #2), and *depth case study and conceptualization* (unclassified cluster).

## Discussion and conclusion

Scientometric analysis provides an interesting and revealing window to understand the evolution and visual history of scholarly work. Based on the rich insights generated by this study, many stories can be told: the emergence, decline and shift in topics that are important in the field of entrepreneurship, the shift in the groups of scholars co-cited as a group, journals that are frequently co-cited as a group, as well as overlooked opportunities and possibly politics in publishing entrepreneurship articles. Given the conflicting findings among previous bibliometric analysis of entrepreneurship research (i.e., entrepreneurship research is “maturing” [10], “diverging and lacking maturity” [11,12,15], “converging” [13]) and the lack of analysis of newer bibliometric data, this study offers a more recent picture of the development of entrepreneurship research that extends and enriches prior bibliometric studies of the field by including multiple units of analysis (i.e., micro = word/term, meso = articles/author, and macro = journal sources) and using a larger, richer and newer dataset (*n* = 3693; 1990–2013), and focusing on articles only (i.e. articles = certified knowledge). In addition, this article offers important methodological contribution to the study of entrepreneurship by introducing three new scientometrics techniques (i.e., topic mapping, time-based and citation-based overlay visualization) as a way of advancing the field and enhancing the validity of the study [28–31]. In fact, this is the *first* article that offers topic mapping and overlay visualization analyses to map the evolution of entrepreneurship research in a single study.

Several key insights emerged from this study that have not been reported or found in prior research, which constitute the contributions in this paper (see Table 2 for a summary of the findings and the observations).

**Table 2:** A summary of scientometric patterns in entrepreneurship research (1990–2013).

Type of scientometric analysis	Summary of findings	Observation
Topic clustering (terms and phrases)	1) An explotion in the number of topics occurred in 2002–2007 (i.e., 28 topic clusters); but the largest explosion was in 2008–2013 period (i.e., 46 topic clusters) (see S1C Fig, Fig 2A and 2B)	Pluralistic topics; some topics weakened, or became a part of other topics, some topics were emerging
	2) 46 topics existed between 1990–2013 (see Table 1, Fig 2A and 2B)	
	3) Topics significantly weakened in 2002–2007 (i.e., "experience and knowledge", "culture") and in 2008–2013 (i.e., "failure", "community and society", "decision making and risk", "competition", "ownership and stakeholders") (see Table 1)	
	4) Topics that became a part of other topics (i.e., 9 topics in 2008–2013) (see Table 1)	
	5) Topics with most significant increase in 2008–2013: "institutional entrepreneurship", "innovation and technology management", "policy and development" (see Table 1, Fig 2A and 2B)	
	6) Newly emerged topics in 2008–2013: "social entrepreneurship", "business ethics", "corporate social responsibility", "leadership", "poverty, norm and tradition", "employment and job creation" (see Table 1)	
	7) 5 topics that stabilized across four periods: "institutional entrepreneurship", "innovation and technology management", "policy and development", "entrepreneurial process and opportunity", "new venture creation"	Stabilization and persistence of five topics
Author co-citation clustering	An explotion in the author co-citation clusters occurred in 2008–2013, with 9 total clusters: "innovation-technology-venture-capital-institution", "economics-innovation-networks", "entrepreneurship-psychology-cognition-sociology-women", "institution-organization-innovation-sociology-network", "social entrepreneurship-narrative-education", "family business-strategy", "strategy-networks-capabilities-exploration", "marketing", "international-entrepreneurship" (see Fig 3, S1D, S1E and S1F Fig).	Diversity of author's co-citation clusters indicating diverse research themes
Journal co-citation clustering	A diverse and complex journal co-citation clustering in X topics" "entrepreneurship-psychology", "management-organizations", "family business", "economics-finance", "technology-innovation", "strategy-management", "international business-entrepreneurship", "marketing-innovation" (see Fig 4)	Pluralistic journal co-citation clusters indicating pluralistic research themes
New topics overlay vizualization	7 new topics emerged (2008 as the average mid point): "institutional entrepreneurship, institutional logic, institutional theory", "social entrepreneurship, narrative, discourse", "poverty", "business ethics", "family business", "internationalization, international entrepreneurship", "global entrepreneurship monitor, use of panel data" (see Fig 5)	Divergence of new topics
Hot (highly cited) topics overlay visualization	9 new hot topics: "institutional work, institutional logic, institutional entrepreneurship", "opportunity discovery and recognition", "international new venture, international business", "entrepreneurial orientation, innovativeness", "cognition, emotion, identity", "top management team", "strategic alliance", "performance, profitability", "depth case study, conceptualization" (see Fig 6)	Divergence of hot topics

First, this study revealed *pluralistic* topics (i.e., 46 topics) that existed in entrepreneurship research between 1990 and 2013. These included *person-centric issues* on entrepreneurship, *performance*, and *new venture creation*, to *family business* and *top management team* (see Table 1). Importantly, in the last period of the study (2008–2013) research in entrepreneurship ‘exploded’ in terms of the number of journal publications as well as the range of topics published (see Table 1). Some topics were less studied or published than others (e.g., failure, competition, decision making and risk); some topics declined for a while and then grew again (e.g., person-centric approach to entrepreneurship and new venture creation); and some others emerged and became more mainstream (e.g., institutional aspect of entrepreneurship). Thus the progression of entrepreneurship as a scientific field is not linear, but highly dynamic; and marked by pluralistic topics that appeared in business and management journals.

Second, this study discovered five topics that were persistent across the 24 years (1990–2013) of the entrepreneurship research (see Table 1, Fig 2 and Fig 3). These were: *institutions and institutional entrepreneurship; innovation and technology management; policy and development; entrepreneurial process and opportunity;* and *new ventures*. These five major topics could be labeled The Pentagon of Entrepreneurship; they are inter-related and form the building blocks of entrepreneurship. Thus my findings support Shane’s [51] reflection, and Busenitz and colleagues’ [14] findings, that there has been a consensus around “opportunity” and “new ventures” in entrepreneurship research, yet also reveal the rise of other topics not reported by these scholars such as *institutional work*, *innovation and technology management*, and *policy and development*.

The findings in this study also support the reflection by Welter and colleagues [52] that research in entrepreneurship has so far focused on ‘wealth creation, high growth, and technology firms’ but question some of their findings (i.e., ‘women/gender’ issue as an understudied domain) where in fact ‘women and gender issue’ has emerged as a frequently studied and published domain in entrepreneurship (see topic cluster #29 in Table 1). The findings also suggested that entrepreneurship research continues to draw from and are published in a diverse number of other disciplines (see Fig 4) and journals (see Fig 5), including psychology, sociology, economics, strategy, international business, and policy and development studies, thus confirming some of the observations of Grégoire and colleagues that the field has *diverging patterns* [12]. This might be driven by scholars publishing strategies that include bringing their ‘non-entrepreneurship’ expertise to contribute to entrepreneurship research (e.g., institutional work in entrepreneurship, the cognition/emotion in entrepreneurship) or employing “novelty seeking strategies” by importing or hybridizing other fields to inform entrepreneurship research [53]. This also suggests that entrepreneurship research “lacks a distinctive character” in that there has been little development of “in house” concepts, theories, perspectives and methods that can be exported to other fields. For example, “effectuation” [54] and “bricolage” [55] are great examples of “in house” concepts that were successfully exported and or adapted to other fields in the social sciences but more efforts are needed to develop them to strengthen the identity of entrepreneurship as a field.

It appears that by employing an “entrepreneurial” lens as a frame, researchers can position almost any research questions and topics to fit in the entrepreneurship field. The *convenience* of extending various disciplines to entrepreneurship coupled with niche-driven research strategy has occurred and may continue to affect the progression of the field. For example, neuroscientists and geneticists have extended their work to entrepreneurship, calling it ‘neuro-entrepreneurship’ [56, 57], while a marriage between operations research and entrepreneurship has given birth to ‘operational entrepreneurship’ [58], and the mix of ethical, social and commercial logic has led to the birth of ‘social entrepreneurship’ [59,60]. In a nutshell, we are witnessing of a “non-paradigmatic” growth of entrepreneurship research as it continues to draw on and mix with other fields to explain and predict entrepreneurial phenomena.

Overall, this study reflects a growing *specialization* and *interdisciplinarity* as the field matures. It offers support for what Cornelius and colleagues [10] call the *signs of maturity* of a field by demonstrating that entrepreneurship research has develop a stable range of topics, an identifiable community of researchers, and increase in *specialization* in the field. This study also supports what Gartner and colleagues [15] call a *highly fragmented* field as scholars bring their own disciplines into the entrepreneurship field. Therefore, the evolution of entrepreneurship as a field of research is not one that is neat or linear but is both convergent and divergent, with a growing consensus on certain topics and the identification of communities of scholars as the field matures and a diversification and interdisciplinarity on the topics and heterogeneity of communities of scholars.

The “struggle for citation”, where scholars compete for recognition from their peers, coupled with the “innovation tournament” through tough journal review processes [61] may have influenced which articles, theories or groups of scholars get cited. Why certain articles, authors or theories become highly cited is beyond the focus of this study. But these processes have given rise to a number of topics that have become highly cited (“hot topics”), including *institutional work*, *opportunity discovery and recognition*, *entrepreneurial orientation*, *cognition/emotion and identity*, *international new venture*, *top management team*, *strategic alliance*, and *performance and profitability*. This may reflect the areas on which leading and highly influential scholars have been and/or are focusing, while indicating the types of topics that the collective community of entrepreneurship scholars and journal editors and reviewers find important.

Consequently, with the emergence of different communities in the field of entrepreneurship as a result of the ‘arrival’ of scholars from other disciplines, *cross-disciplinary* research can be better promoted as a more fruitful avenue to push research in entrepreneurship forward and to break new grounds in the field. As this study shows, research on the *everydayness* of entrepreneurship (i.e., those classified as non-heroic, non-messianic and non-growth- or technology- oriented) remains scant in the literature. On this note, studying entrepreneurship in specific contexts may offer new ways of advancing the entrepreneurship field. As this scientometric study shows, entrepreneurship research has not given sufficient attention on a number of specific contexts as a way of advancing the field. One of these is the *spirituality and religiosity* aspects in entrepreneurship [62]. While most entrepreneurship research takes a secular perspective, the role of spiritual and religious values and other normative values such as ideology [63] could be new and interesting perspectives and contexts to study entrepreneurial cognition and action. In the context of developing economies (e.g., Africa) and Asia (e.g., China or Malaysia or Mexico), for instance, it is public knowledge that entrepreneurial decision making are often shaped by Confucianism, Islam, Christianity or even folk beliefs as well as local politics. This research direction may attract scholars from the field of religion and politics and or collaborations between scholars in entrepreneurship and religion and politics to form a cross-disciplinary research with entrepreneurship.

The other is sustainable entrepreneurship [64–66]. *Sustainability* is another relatively new context to entrepreneurship, and involves economic and non-economic aspects as well as collective action and political will in facilitating sustainable business practices–thus providing new theoretical perspectives to advance our understanding of entrepreneurship as ecologically-friendly behavior. Future research can promote more cross-disciplinary research that brings in scholars and expertise from environmental, energy and material science and fuse them with entrepreneurship. The impact of entrepreneurial activities on the Earth’s geology and ecosystems (e.g., use of chemicals in modern agriculture enterprises), entrepreneurial efforts to promote versus discourage sustainable entrepreneurship (e.g., environmental activism turned sustainable ventures; fast fashion industry or artificial intelligence-driven enterprises as a driver of unsustainable world), to the relationship between new geopolitical orders and sustainable enterprises (e.g., China’s One Belt One Road and its influences on economic and environmental sustainability in various parts of the world).

Next, studies of *developing and less/least developed market* as a context remain marginalized in mainstream research on entrepreneurship. Many of the ‘jamu’ or ‘sari sari’ or floating market entrepreneurs (a common phenomenon in Indonesia, Philippines, and Thailand respectively [67,68]) do not pursue wealth creation but treat entrepreneurship as a means of survival and pursuing a simple lifestyle. We know little about these phenomena but they could enrich and extend entrepreneurship theories. In short, I argue that ‘context is king’ in future entrepreneurship research. Last but not least, the dearth of ‘replication studies’ in entrepreneurship found in this scientometrics study suggests that more research is needed to test and re-test prevailing assumptions and generalizations in the field.

Finally, as a caveat, I acknowledge that the present study reflects only one of many alternative interpretations of the development of the field of entrepreneurship. The research design in this study may have excluded certain or some publications from certain journals (e.g., engineering, law, public policy journals). Thus, future research can examine the bibliographic materials of such entrepreneurship-specific journals as the *Journal of Business Venturing*, *Entrepreneurship Theory and Practice*, and the *Strategic Entrepreneurship Journal* from their first issue until the most recent issues, to see how they collectively “tell a story” about the evolution of the field. A cross journal comparison of them will show how the topic evolution, co-citation relations, or new and hot topics may differ across each journal. Another alternative would be to study how entrepreneurship research evolves within a specific area or a ‘local analysis’, such as business and management, versus the global categories or ‘global analysis’ that include science, engineering, arts and other social sciences fields. This would offer a richer understanding of how scholars in different fields conduct entrepreneurship-related research. Another possible area of research would be to see the knowledge diffusion of theories, concepts or ideas (e.g., effectuation, bricolage, entrepreneurial orientation) from entrepreneurship-specific journals to non-entrepreneurship and the broader management and organization journals. Future studies could also take a more inclusive approach by including non-journal outlets, such as popular and textbooks, conference proceedings, and practitioner-oriented journals, to understand how entrepreneurship diffuses throughout various channels and fields. Last but not least, future studies could adopt alternative techniques, from topic modeling to computer-aided text analysis and computational linguistics, to test and explore patterns in the publications in entrepreneurship.

## Supporting information

S1 Fig Topic clusters 1990–1995 (n = 121). (TIF)

S2 Fig Topic clusters 1996–2001 (n = 262). (TIF)

S3 Fig Topic clusters 2002–2007 (n = 866). (TIF)

S4 Fig Author co-citation 1990–1995. (TIF)

S5 Fig Author co-citation 1996–2001. (TIF)

S6 Fig Author co-citation 2002–2007. (TIF)
