# Supplementary figures and images for: Mapping the evolution of entrepreneurship as a field of research (1990–2013): A scientometric analysis
Source: PLoS One. 2018 Jan 4;13(1):e0190228. doi: 10.1371/journal.pone.0190228 (PMC5754054; doi:10.1371/journal.pone.0190228)

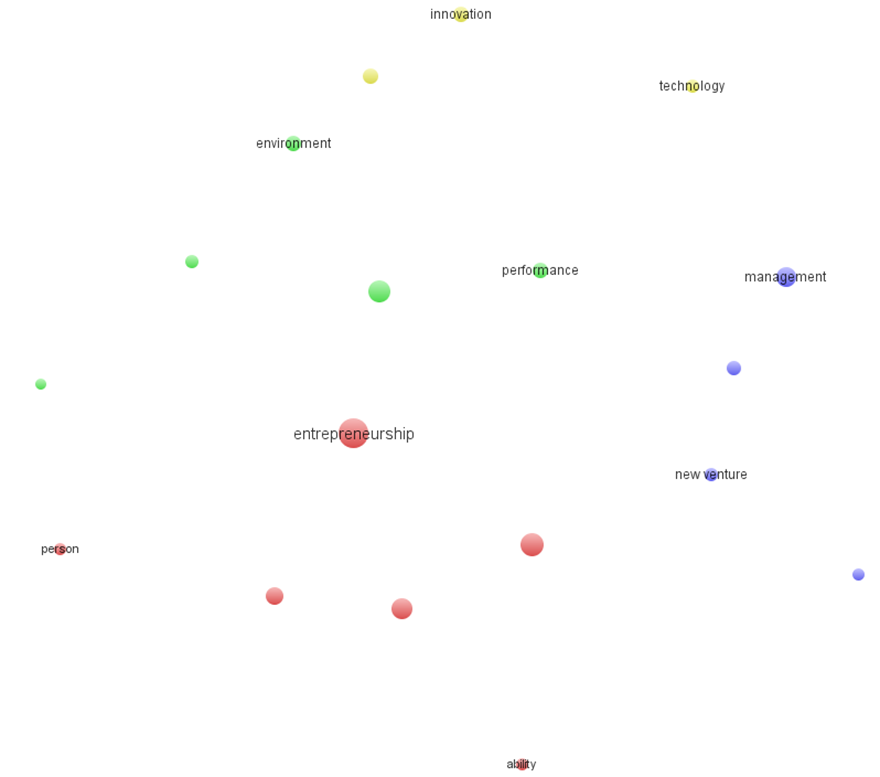

Supplement: S1 Fig — (TIF) [file pone.0190228.s001.tif]

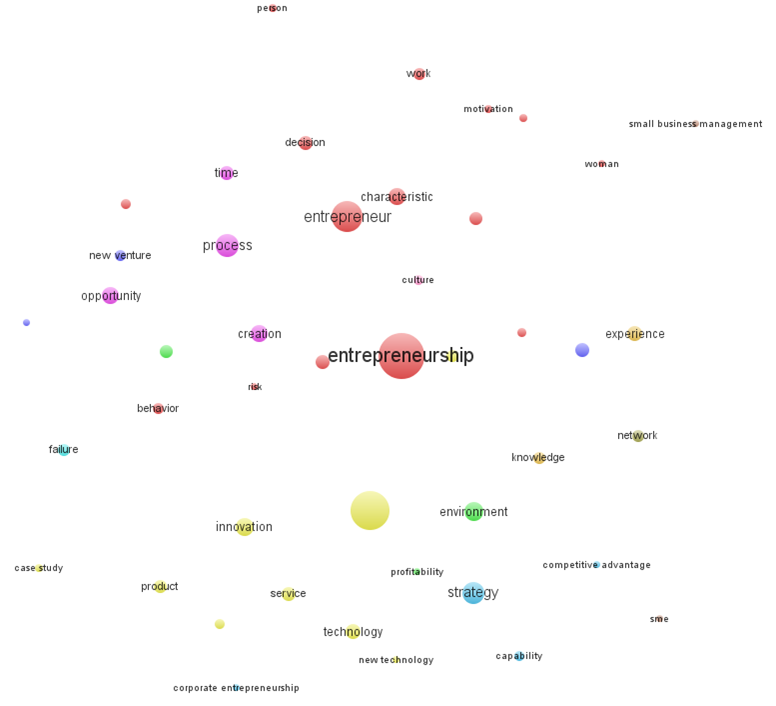

Supplement: S2 Fig — (TIF) [file pone.0190228.s002.tif]

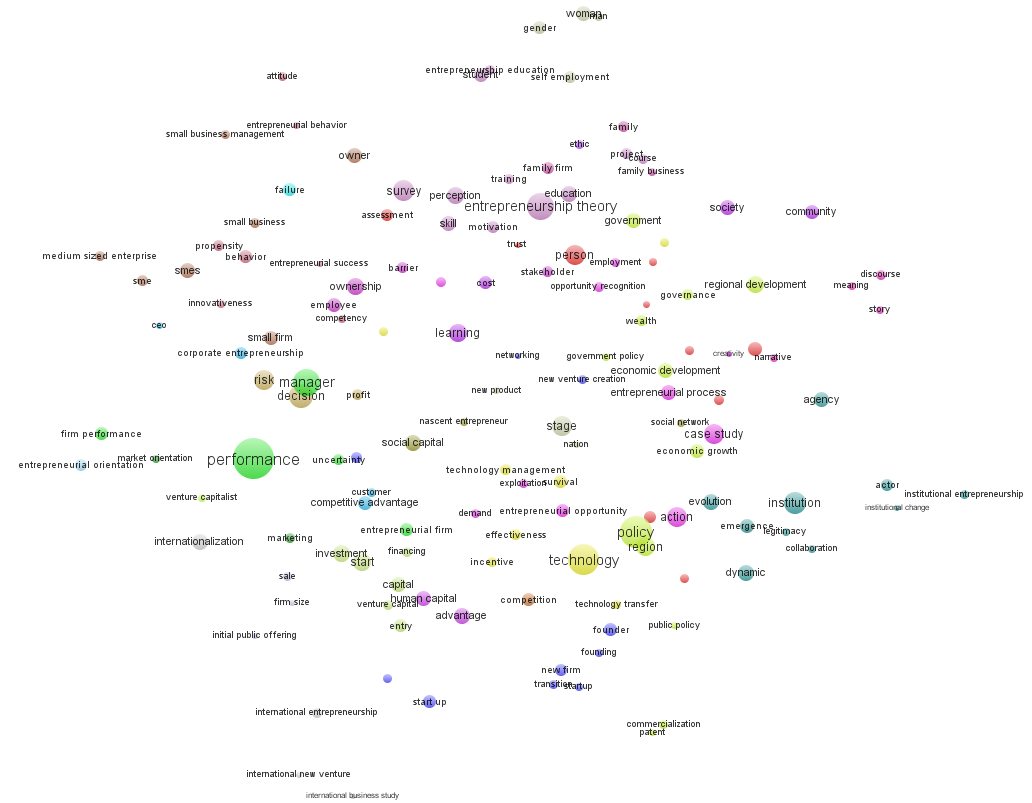

Supplement: S3 Fig — (TIF) [file pone.0190228.s003.tif]

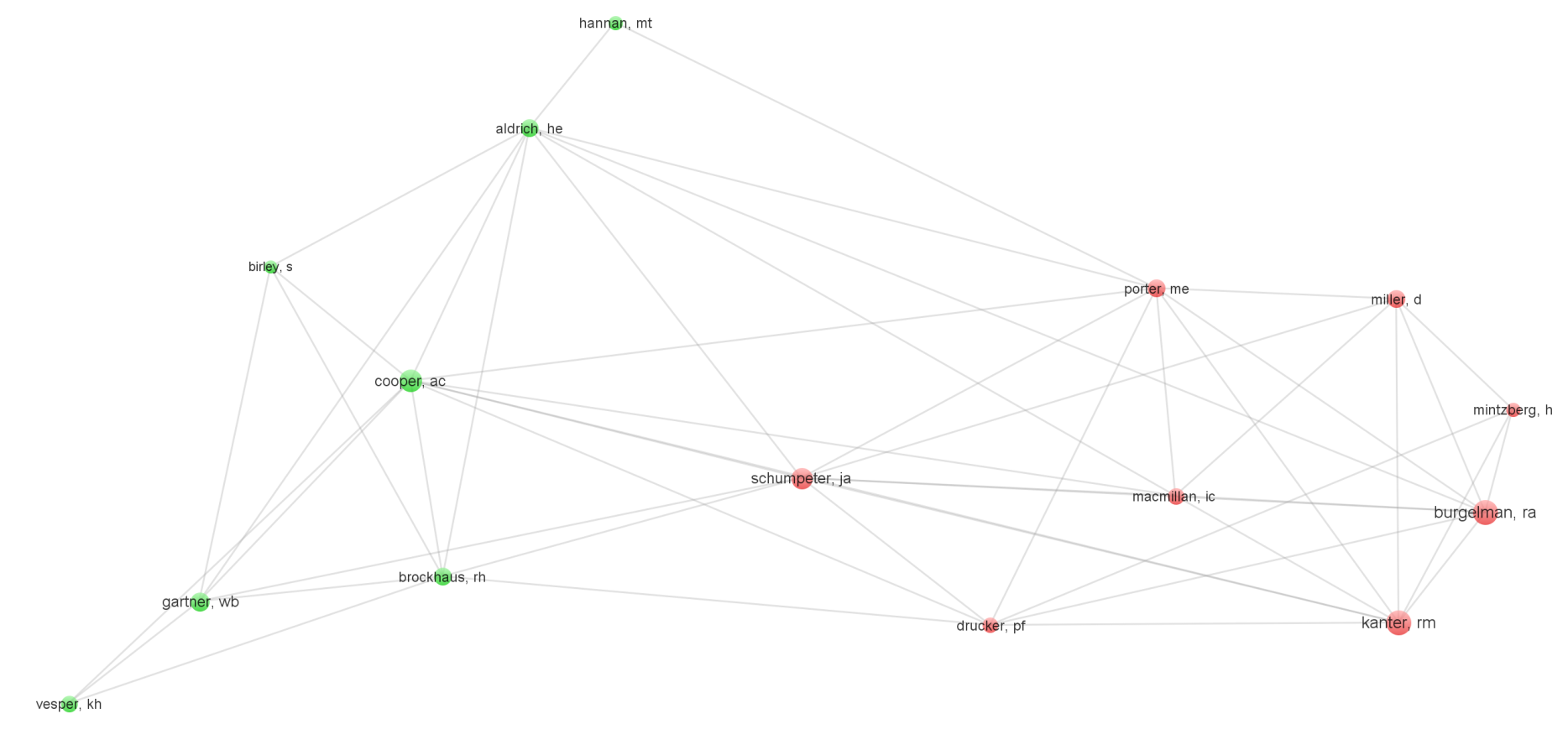

Supplement: S4 Fig — (TIF) [file pone.0190228.s004.tif]

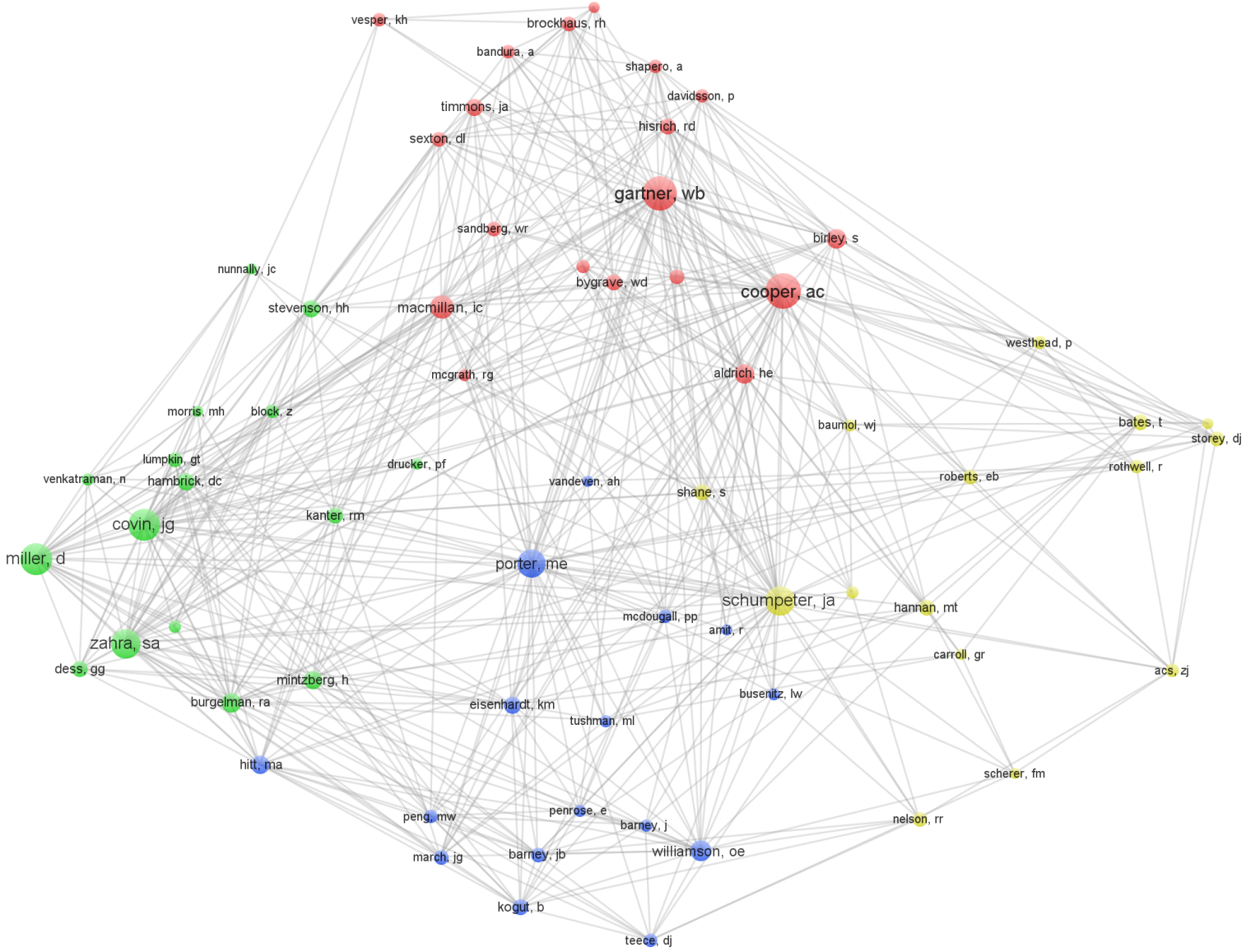

Supplement: S5 Fig — (TIF) [file pone.0190228.s005.tif]

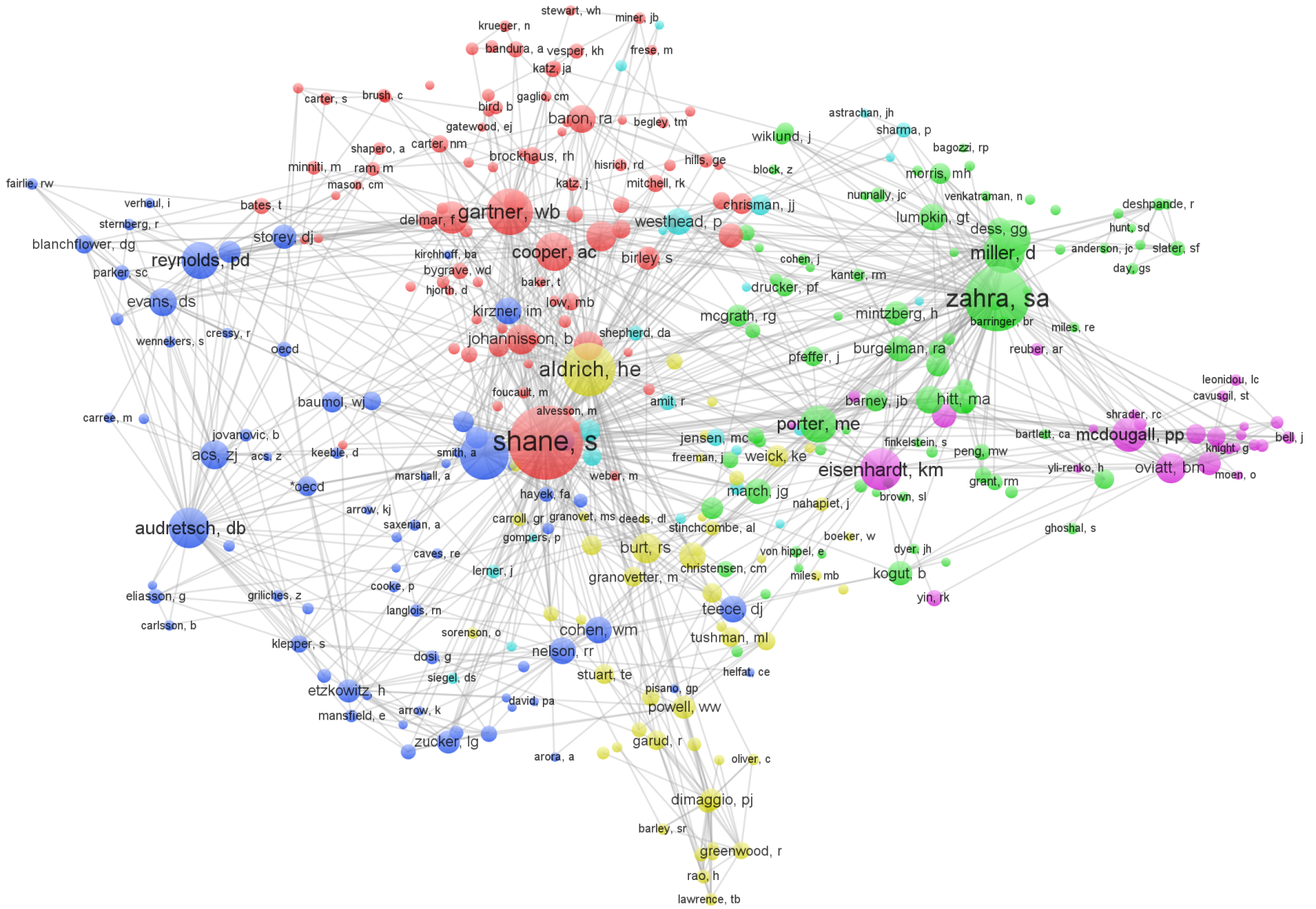

Supplement: S6 Fig — (TIF) [file pone.0190228.s006.tif]
